# Epigallocathechin gallate, polyphenol present in green tea, inhibits stem-like characteristics and epithelial-mesenchymal transition in nasopharyngeal cancer cell lines

**DOI:** 10.1186/1472-6882-12-201

**Published:** 2012-10-30

**Authors:** Chien-Hung Lin, Yao-An Shen, Peir-Haur Hung, Yuan-Bin Yu, Yann-Jang Chen

**Affiliations:** 1 Institute of Clinical Medicine, National Yang-Ming University, Taipei, Taiwan; 2 Department of Pediatrics, Zhongxing Branch, Taipei City Hospital, Taipei, Taiwan; 3 Institute of Biochemistry and Molecular Biology, National Yang-Ming University, Taipei, Taiwan; 4 Department of Internal Medicine, Ditmanson Medical Foundation Chia-yi Christian Hospital, Chia-yi, Taiwan; 5 Division of Hematology and Oncology, Department of Medicine, Taipei Veterans General Hospital, Taipei, Taiwan; 6 Department of Pediatrics, Renai Branch, Taipei City Hospital, Taipei, Taiwan; 7 Department of Life Sciences and Institute of Genome Sciences, National Yang-Ming University, Taipei, Taiwan

**Keywords:** Epigallocathechin gallate, Nasopharyngeal carcinoma, Cancer stem cells, Stem cells, Epithelial-mesenchymal transition

## Abstract

**Background:**

Previous studies have demonstrated that the consumption of green tea inhibits the growth of various cancers. Most cancers are believed to be initiated from and maintained by a small population of cancer stem-like cells (CSC) or tumor-initiating cells (TIC) that are responsible for tumor relapse and chemotherapeutic resistance. Although epigallocathechin gallate (EGCG), the most abundant catechin in green tea, has been reported to induce growth inhibition and apoptosis in some cancer cells, its effect on CSC is undefined. In this study, we enriched CSC by the sphere formation, and provided an efficient model for further experiments. Using this method, we examined the effects of EGCG regulating the nasopharyngeal carcinoma (NPC) CSC and attempted to elucidate the possible mechanisms.

**Methods:**

NPC TW01 and TW06 cell lines were enriched by sphere formation and characterized their phenotypical properties, such as invasion capacity, epithelial-mesenchymal transition (EMT) and gene expression were analyzed by quantitative real-time reverse transcription polymerase chain reaction (q-RT-PCR). EGCG-induced growth inhibition in the parental and sphere-derived cells was determined by MTT and bromodeoxyuridine (BrdU) assay. EGCG-induced apoptosis was analyzed by flow cytometry with Annexin V and PI staining. The effects of EGCG on sphere-derived cell tumorigenicity, migration and invasion were determined by soft agar assay, wound healing, and cell invasion assay. The alternation of protein expression regulated by EGCG on these sphere-derived cells was assessed by immunofluorescence staining and western blot.

**Results:**

NPC sphere-derived cells grown in serum-free non-adherent culture showed increased expression of stem cell markers and EMT markers compared to parental cells grown in conventional culture. Although EGCG induced growth inhibition and apoptosis in the parental cells in a dose-dependent manner, it was not as effective against spheres. However, EGCG potently inhibited sphere formation and can eliminate the stem cell characteristics of NPC and inhibit the epithelial-mesenchymal transition (EMT) signatures.

**Conclusions:**

Overall, these findings show that NPC cells with sphere formations possess the properties of CSC. Using this model, we found that EGCG regulated NPC CSC, their self-renewal capacity, and inhibited their invasive characteristics. It supports the pivotal role of EGCG as a dietary compound targeting NPC and may decrease recurrence and metastasis in nasopharyngeal carcinoma cells.

## Background

Nasopharyngeal carcinoma (NPC) arises from the nasopharyngeal epithelial cells with high prevalent rates in Southeast China and Taiwan. According to the World Health Organization (WHO) histological classification, NPC is categorized into three groups: keratinizing squamous cell carcinoma (SCC, type I), nonkeratinizing carcinoma (NKC, type II) and undifferentiated carcinoma (UC, type III) [1]. NPC has high metastatic potential with frequent initial dissemination to regional lymph nodes, and distant metastases at diagnosis, and this underlies the majority of NPC-related death [2,3]. Advanced-stage NPC might have chemotherapy resistance and radio-resistance. The prognosis remains poor in a significant number of NPC patients with relapse or metastatic diseases.

Evidences support the hypothesis that tumors contain small subpopulations of cells called cancer stem-like cells (CSC) or tumor-initiating cells (TIC) [4,5]. These cells are generally thought of as self-renewing cells that are able to reinitiate a tumor for several generations and can give rise to a spectrum of differentiated cells. CSCs are able to proliferate and self-renew extensively because of their ability to express anti-apoptotic and drug-resistant proteins, thus sustaining tumor growth [6]. Presumed cancer stem-like cells had been proposed in nasopharyngeal tumors by side population and surface markers [7,8].

To eradicate NPC and prevent recurrence, it is imperative that NPC CSC should be specifically and efficiently inhibited. In addition, CSC undergoing metastasis often expresses epithelial-to-mesenchymal transition (EMT) and the high EMT markers have been observed in dissemination of carcinoma cells from primary epithelial tumors [9]. The interaction between stemness characteristics and EMT expression has advanced results in recent studies. Researches have shown that EMT acquires CSC properties, increases cancer cell tumorigenicity and shows a crucial link to metastasis [10-12].

One assay for enriched and characterized stem cells is based on the sphere-generated cells growing in serum free suspension. These cells may represent a tumor-initiating subpopulation with an ability of self-renewal, and proliferate unlimitedly [13]. We demonstrated that the sphere-derived cells of NPC are more invasive, chemoresistant, and express anti-apoptosis and EMT genes compared to parental monolayer cells and these served as a model for designing experiments.

The cancer preventive effects of green tea are widely supported by results from epidemiological, cell culture, animal and clinical studies. This property has mainly been attributed to the most prevalent tea polyphenol, epigallocathechin gallate (EGCG), which has potent capacity in inhibiting cancer growth and inducing apoptosis in various cancers by several mechanisms. It causes cell cycle deregulation and apoptosis of cancer cells through NF-kappa B inhibition and activation of caspases [14,15]. It inhibited expressions of Bcl-2 and Bcl-XL and induced expressions of Bax, Bak and Bcl-XS [16,17]. In addition, EGCG inhibited angiogenesis and metastasis through repressing the expression of vascular endothelial growth factor (VEGF), and matrix metalloproteinases (MMP)-2 and MMP-9 in prostate cancer [18]. It is also an important preventive agent for declining cancer incidence, and has synergistic effects on cancer cell responses to conventional chemotherapy [19-21].

Recent studies have shown that some dietary compounds have potential to act against CSC through several regulatory mechanisms [22-24]. However, EGCG involved in possible mediated molecular mechanisms and therapeutic targets in regulating CSC has been largely unexplored.

This study investigated if EGCG can target NPC CSC by using sphere-derived cells, and realized the underlying molecular mechanisms.

## Methods

### Cell culture

#### Parental monolayer cell culture

NPC TW01 (WHO type I, keratinizing squamous cell carcinoma) and TW06 (WHO type III, undifferentiated carcinoma) cell lines established from Taiwanese were cultured in 10cm^2^ dishes with Dulbecco’s Modified Eagle Medium (DMEM) (Invitrogen Carlsbad, CA) and 10% FBS (BIOIND, Kibbutz Beit Haemek, Israel), 1% sodium pyruvate (BIOIND), 1% penicillin, streptomycin, amphotericin (PSA, BIOIND) and 1% Non-Essential Amino Acids (NEAA, BIOIND). The cells were incubated at 37°C in a humidified atmosphere of 5% CO2.

#### Non-adherent culture

TW01 and TW06 parental cells were seeded non-adhesively in 6-well culture dishes coated with thin agarose at a density of 2 × 10^4^/mm^3^ in serum-free DMEM/F12 medium (Invitrogen). Each treatment was carried out in triplicates. The culture medium was changed every other day until sphere formation. After 7 to 10 days, the spheres were collected by filtration through a 70 μm mesh for the following experiments.

#### RNA extraction and quantitative real-time RT-PCR

The total RNA was isolated with Trizol reagent (Invitrogen). First-strand cDNA was reverse transcribed (RT) according to the manufacturer protocols. Relative levels of mRNA were determined by qPCR using a real time PCR system. Several stemness genes and epithelial-mesenchymal transition markers were analyzed. The primer sequences used for quantitative real-time RT-PCR are shown in Table 1. GAPDH was the endogenous reference. cDNA was subjected to PCR for 35 cycles of 94°C for 30 s, 55°C for 30 s, and 72°C for 45 s. q-PCR was performed using an ABI PRISM® 7900HT system (Applied Biosystems, Foster City, CA).

**Table 1:** The primer sequences for q-RT PCR used in this study

**Gene**	**Sequence**
GAPDH	Forward : 5^′^-ACGGGAAGCTCACTGGCATGG-3^′^
	Reverse : 5^′^-GGTCCACCACCCTGTTGCTGTA-3^′^
Sox-2	Forward : 5^′^-CGAGTGGAAACTTTTGTCGGA-3^′^
	Reverse: 5^′^-TGTGCAGCGCTCGCAG-3^′^
Oct-4	Forward: 5^′^-GTGGAGAGCAACTCCGATG-3^′^
	Reverse: 5^′^-TGCTCCAGCTTCTCCTTCTC-3^′^
c-Myc	Forward: 5^′^-GGAACGAGCTAAAACGGAGCT-3^′^
	Reverse: 5^′^-GGCCTTTTCATTGTTTTCCAACT-3^′^
Klf-4	Forward: 5^′^-CCGCTCCATTACCAAGAGCT-3^′^
	Reverse: 5^′^-ATCGTCTTCCCCTCTTTGGC-3^′^
E-Cad	Forward: 5^′^-TGCCCAGAAAATGAAAAAGG-3^′^
	Reverse: 5^′^-GTGTATGTGGCAATGCGTTC-3^′^
Vim	Forward: 5^′^-GAGAACTTTGCCGTTGAAGC-3^′^
	Reverse: 5^′^-GCTTCCTGTAGGTGGCAATC-3^′^
Snail	Forward: 5^′^-CTTCCAGCAGCCCTACGAC-3^′^
	Reverse: 5^′^-CGGTGGGGTTGAGGATCT-3^′^
N-Cad	Forward: 5^′^-AGGGTGGACGTCATTGTAGC-3^′^
	Reverse: 5^′^-CTGTTGGGGTCTGTCAGGAT-3^′^
Twist	Forward: 5^′^-CGAGTCCGCAGTCTTACGAG-3^′^
	Reverse: 5^′^-TCTGGAGGACCTGGTAGAGG-3^′^

#### Cell proliferation assay by MTT and BrdU assay

The effect of EGCG on proliferation of NPC TW01, TW06 parental and sphere-derived cells was examined by MTT [3-(4, 5-dimethylthiazol-2-yl)-2, 5-diphenyltetrazolium bromide] assay. Cells (2.5 × 10^3^ per well) were seeded in 96-well plates and allowed to grow for 24 h. Then EGCG or DMSO (vehicle control) was added after plating onto adherent cells at specified concentrations. Each treatment was triplicated. After 72 h, 20 μl of MTT solution (5 mg/ml; Sigma, St. Louis, MO) was added to each well and incubated for 4 h at 37°C. The MTT formazan crystal was then dissolved in DMSO, and the absorbance was measured by a microplate reader (Bio-Rad 680, Bio-Rad Laboratories, Hercules, CA) at a wavelength of 570 nm.

Cell proliferation was also determined by bromodeoxyuridine (BrdU) incorporation analysis (Roche Applied Science, Mannheim, Germany). Briefly, cells were plated in 96-well plates (2.5 × 10^3^ per well) for 24 h and BrdU incorporated in the newly-synthesized DNA of EGCG-treated or vehicle was quantified after 72 h. After labeling with BrdU (10 μM) for 2 h, cells were fixed, incubated with anti-BrdU-peroxidase (10 μl/well) for 1 h and 30 min, and followed by the addition of trimethyl benzidine substrate to measure the amount of incorporated BrdU. Absorbance of each well was measured at wavelength of 450 nm.

#### Apoptosis assessed by flow cytometry

TW01 parental and sphere-derived cells were treated after plating in the monolayer with different concentrations of EGCG for 72 h respectively. Cells were washed twice with cold PBS and then resuspended in 1X Binding Buffer at a concentration of 1 × 10^6^ cells/ml. Transfer of 100 μl of solution (1 × 10^5^ cells) to a 5 ml culture tube was performed and 5 μl of FITC Annexin V and 5 μl propidium iodide (PI) (BD Pharmingen, San Diego, CA) were then added. The cells were gently vortexed, and incubated for 15 min at RT (25°C) in the dark. A 400 μl of 1X Binding Buffer was added to each tube. Analysis was performed by FC500 flow cytometer (Beckman Coulter, Brea CA) to identify the subpopulations of the apoptosis cells within 1 hr.

#### Soft agar clonogeneic assay

The bottom of each well (35 mm) of 6-well culture dish was coated with 2 ml agar mixture (DMEM, 10% [v/v] FCS, 0.6% [w/v] agar). After the bottom layer solidified, 2 ml top agar-medium mixture (DMEM, 10% [v/v] FCS, 0.3% [w/v] agar) containing 2 × 10^4^ sphere-derived cells with different concentrations of EGCG was added and incubated at 37°C for 2 weeks. At the end of the incubation period, the number of colonies was counted using microscope after staining with the crystal violet.

#### Wound healing assay

NPC TW01 parental and sphere-derived cells were seeded in 6-well plates and grown to confluence, and then added with EGCG. Monolayers were scraped with a micropipette and photographed at the beginning of the assay at 0 h and at 24 h in respective conditions.

#### Cell invasion assay

Cultrex® 96 Well Collagen IV Cell Invasion Assay (Trevigen Inc. Gaithersburg, MD) was used according to the manufacturer instructions. Briefly, cells were cultured to 80% confluence, serum starved over-night, then harvested and re-suspended to a density of 5 × 10^4^ cells/ml in serum-free medium. Cell suspension of 50 μl was added to the top chamber with and without EGCG, and 150 μl of media with FBS was added to the lower well. The top invasion chamber was coated with a membrane of 50 μl BME (basement membrane extract) solution. The chambers were incubated at 37°C in a humidified environment for 24 h. The following day, cells invading the lower wells were detected by calcein-AM staining followed by fluorescence measurement on a microplate reader at 485 nm excitation and 520 nm emissions.

#### Immunofluorescence staining

Cells were cultured on sterile glass coverslips in 6-well plates and fixed in 4% paraformaldehyde. After washing with PBS, the cells were permeabilized using 0.1% Triton X-100 solution and blocked with 10% BSA. The slides were incubated overnight at 4°C with the primary antibodies, followed by incubation with the fluorescently-labeled secondary antibodies at room temperature for 1 h. Slides were counterstained with 4′-6-diamidino-2-phenylindole (DAPI) and analyzed under a fluorescent microscope (Leica DM 4000B).

#### Western blot analysis

Western blot was performed to detect proteins involved in EMT (E-cadherin, Snail, and Vimentin). Cells were lysed in a RIPA buffer containing a 1X protease inhibitor cocktail, and protein concentrations were determined using the Bradford assay (Bio-Rad, Philadelphia, PA). Proteins were separated by 10%-12.5% SDS/PAGE and transferred to membranes (Millipore, Bedford, MA) at 55 V for 4 h at 4°C. After blocking in 5% non-fat dry milk in TBS, the membranes were incubated with primary antibodies at 1:1000 dilution in TBS overnight at 4°C, washed 3 times with TBS-Tween 20, and then incubated with secondary antibodies conjugated with horseradish peroxidase at 1:5000 dilution in TBS for 1 h at room temperature. Membranes were washed again in TBS-Tween 20 3 times at room temperature. Protein bands were visualized on X-ray film using an enhanced chemiluminescence detection system. The primary antibodies against E-cadherin and Vimentin were purchased from Sigma-Aldrich (St Louis, MO). Snail from Abcam (Cambridge, MA) and used at a dilution of 1:1000.

#### Statistical analysis

Data are expressed as the mean ± SD from a minimum of 3 separate experiments. The differences between 2 groups were analyzed by the Student *t*-test. The differences among 3 groups were analyzed by one or two way ANOVA, statistical significance was considered as *p*<0.05. Statistical analyses were performed with SPSS for Windows version 14.0.

We had ethical approval. Our experimental research performed with the approval of an appropriate ethics committee. Our research carried out *in vitro*, not on humans and not on animals.

## Results

### NPC Cells with ability to form spheres acquired features of CSC and underwent EMT

**Figure 1:**
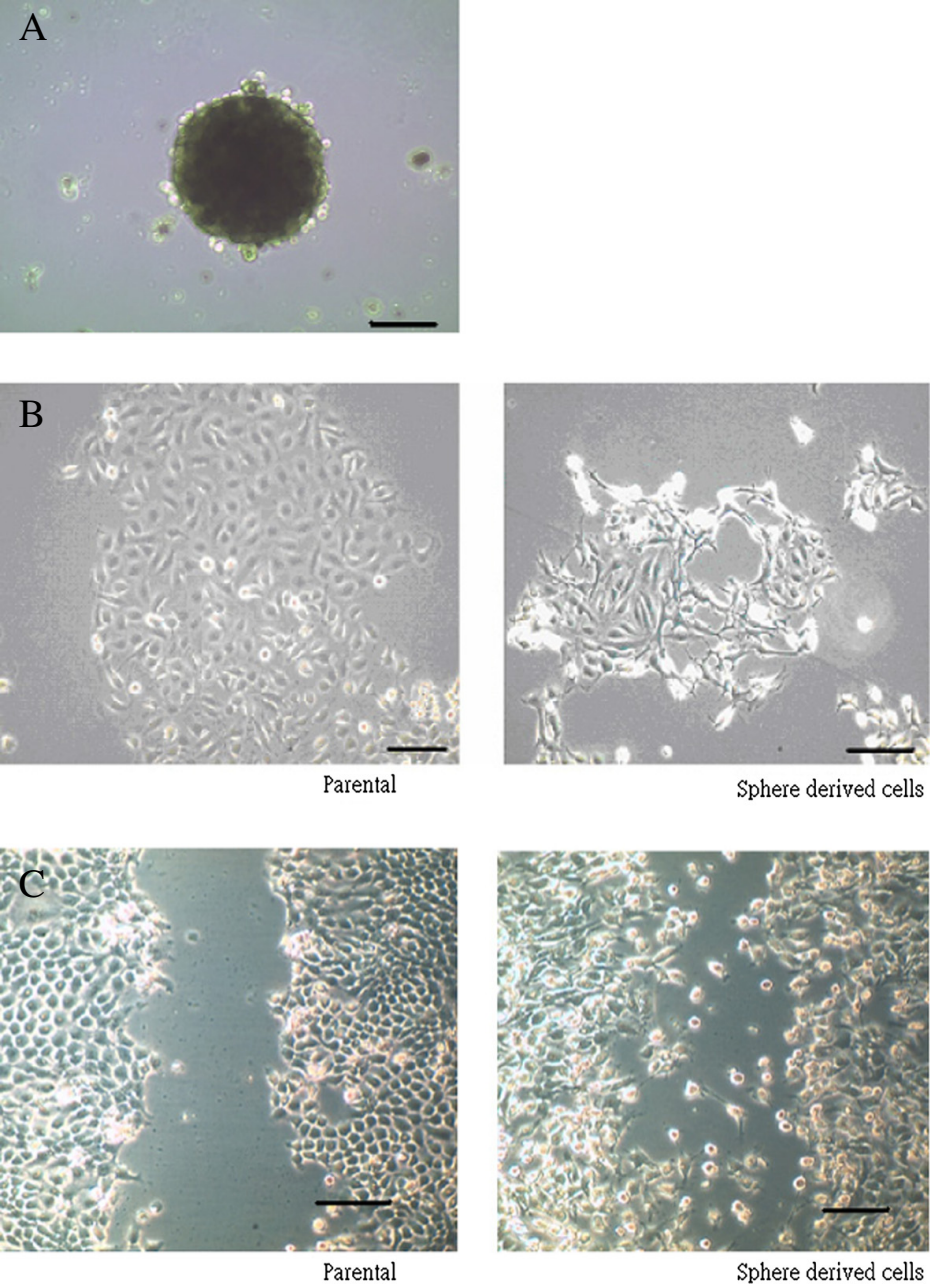
**NPC sphered cells showed different properties compared to parental cells.** (**A**) NPC TW01 sphere grew in serum free non-adherent condition (**B**) Different cell shape of TW01 parental and sphere-derived cells were noted when cultured adherently in 10% serum for 48 h. Sphered cells present in mesenchymal shape. (**C**) Sphere-derived cells present more powerful migratory behavior after 12 h of scraping (Scale bars show 100 μm).

**Figure 2:**
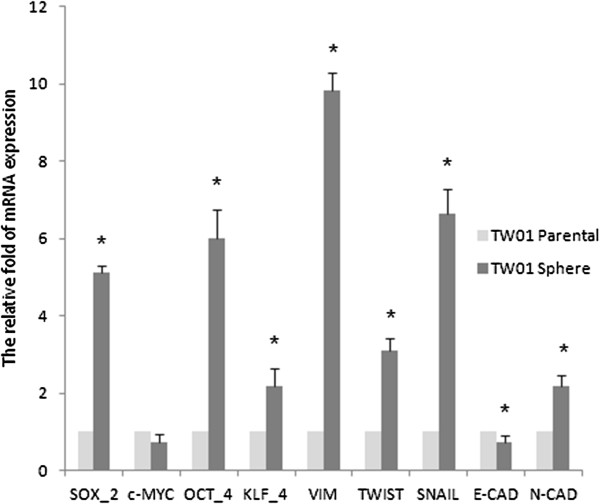
**mRNA expressions of stemness genes and EMT markers analyzed by q-RT-PCR.** The amounts of Sox-2, Oct-4, Klf-4 and EMT markers Vimetin, Twist, Snail and N-cad were increased in TW01 sphere-derived cells as compared to parental cells. The internal control is housekeeping gene-GAPDH and every gene expression of sphere-derived cells was normalized with parental cells. Data represent mean ± SD. * means significantly different from respective controls, *p* < 0.05.

First, we investigated NPC cell lines containing cells with self-renewal that form tumor spheres, and have the ability to migrate, invade, and express the EMT phenotype (Figure 1). NPC cells showed increased expression of stem cell markers Sox-2, Oct-4, and Klf-4 in sphere-derived cells grown in serum-free non-adherent culture compared to parental cells grown in conventional culture. In addition, the expression of mRNA of the transcription factors involved in EMT, such as Twist, Snail, and mesenchymal makers, such as Vimetin and N-cadherin were also increased in sphere-derived cells compared to the parental cells. However, a decreased expression of E-cadherin in sphere-derived cells was detected (Figure 2).

### Effects of EGCG on TW01, TW06 growth, and apoptosis

**Figure 3:**
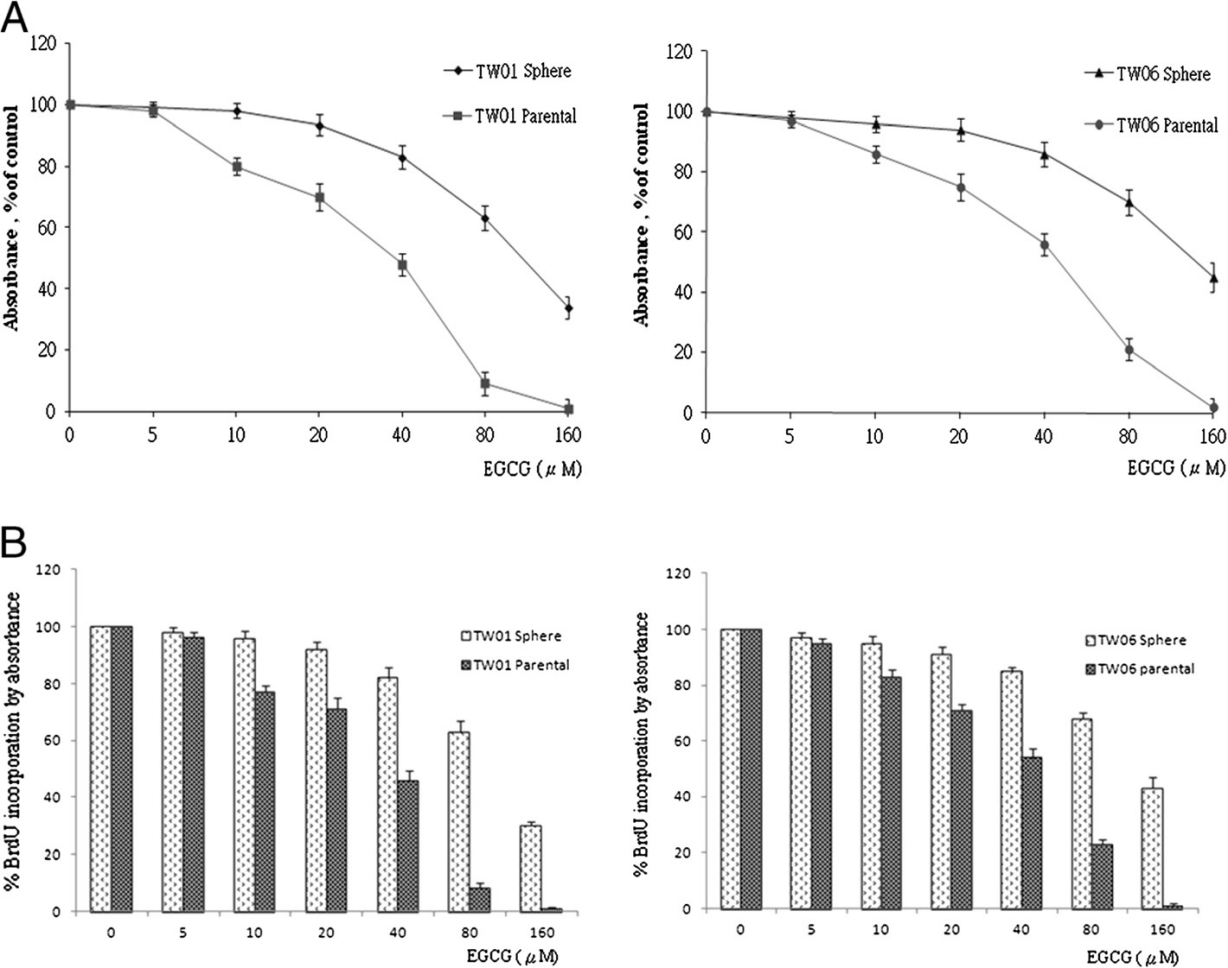
**Effect of EGCG on NPC cell proliferation.** EGCG had effects on cell proliferation and induced growth inhibition of NPC parental and sphere-derived cell in a concentration dependent manner. However, sphere-derived cells showed relative resistance to EGCG (μM) compared to parental cells. (**A**) MTT assay and (**B**) BrdU assay. Both assays showed similar results.

The proliferation-inhibition effects of EGCG with different concentrations in NPC TW01 and TW06 cell lines were evaluated by MTT assay and BrdU assay. Both results showed EGCG-induced inhibition of TW01 and TW06 proliferation in a concentration dependent manner (Figure 3). However, sphere-derived cells showed more resistance to the EGCG-inhibition effect compared to parental cells, and were less effective in inducing growth inhibition.

**Figure 4:**
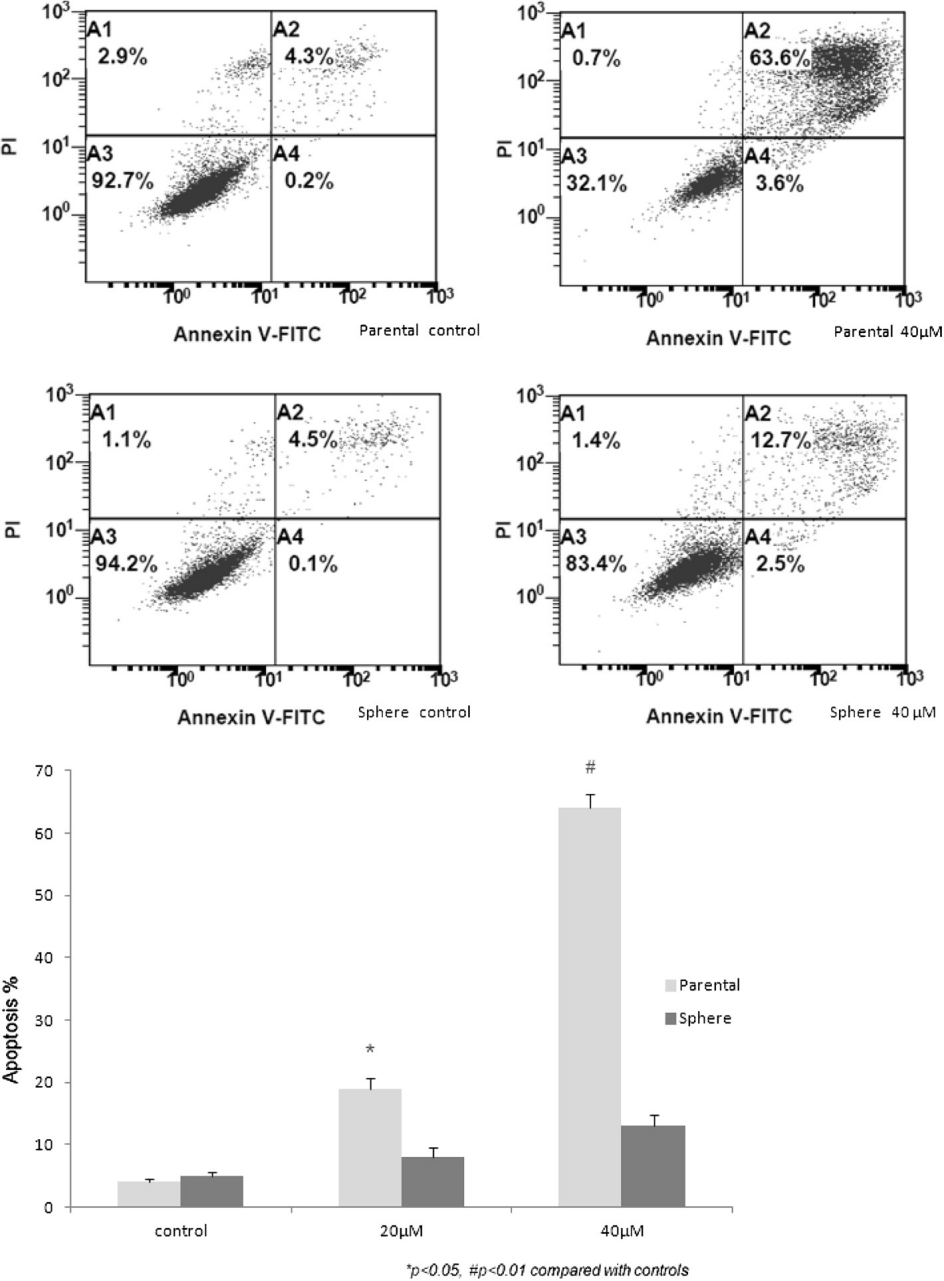
**Apoptosis effect of EGCG on NPC cells.** The upper panel shows the apoptosis frequency of TW01 parental cell increased from 4.3% up to 63.6% after 40μM EGCG treatment, while only up to 12.7% is noted in the sphere-derived cells. The lower panel shows EGCG-induced TW01 parental cell apoptosis was significantly correlated with a dose-dependent trend but was not as effective in TW01 sphere-derived cells.

The apoptosis effect modulated by EGCG was detected by flow cytometry with Annexin V and PI double staining. The results showed increased apoptotic activity in TW01 parental cells treated with 40 μM EGCG for 72 h but this was not apparent in TW01 sphere-derived cells (Figure 4). As compared to the control, EGCG-induced TW01 parental cell apoptosis significantly correlated to dose-dependent trends, however, this did not occur in TW01 sphere-derived cells.

### Inhibition on NPC sphere-derived cell colony formation, migration, and invasion by EGCG

**Figure 5:**
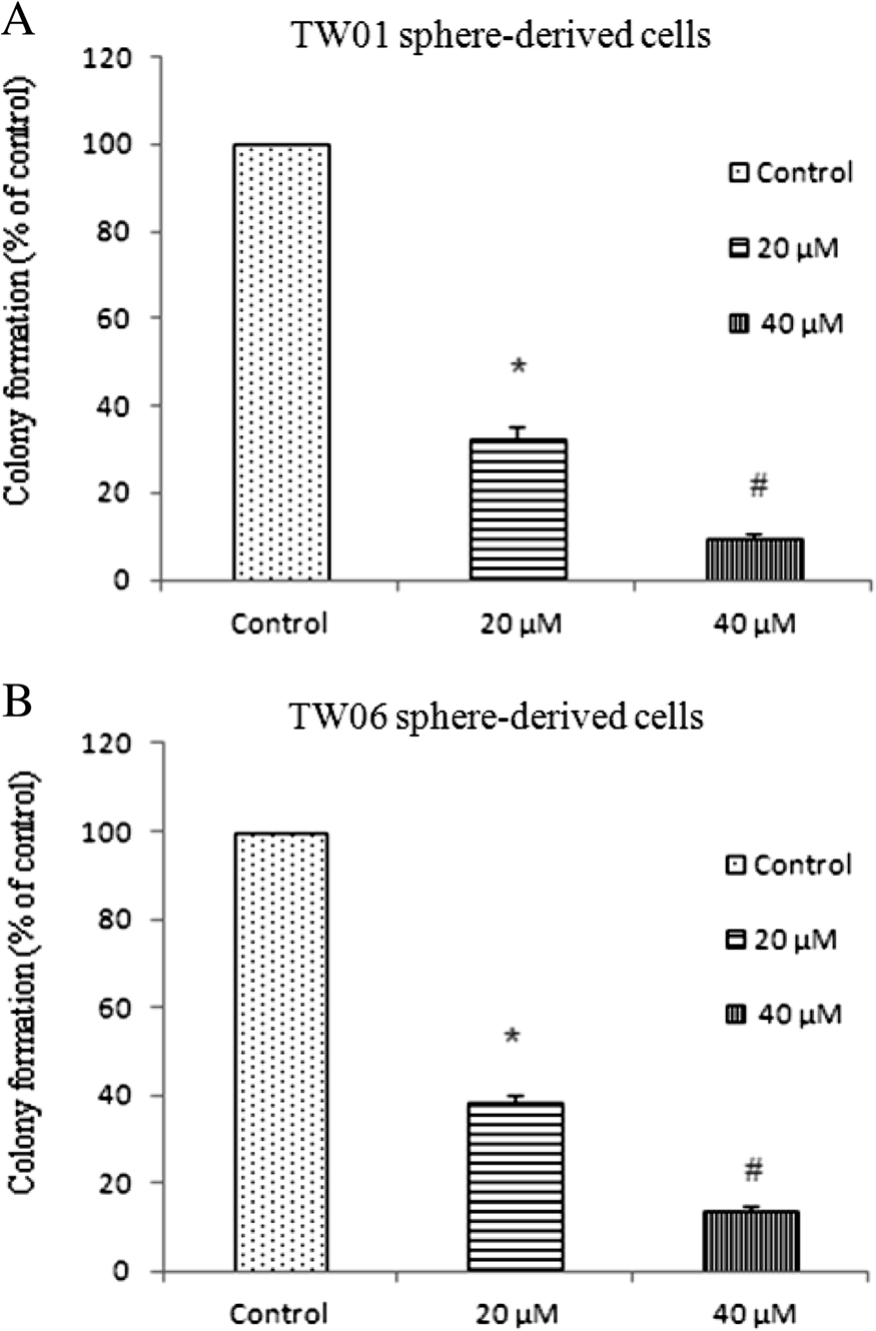
**Inhibition of colony formation of EGCG in a dose-dependent manner.** Both TW01 and TW06 sphere-derived cells were treated with 20 μM or 40 μM EGCG and performed colony formation assay. Decreasing colony number are noted when EGCG concentration increasing. Data represent mean ± SD. * or # means significantly different from respective controls*, p* < 0.05.

TW01 and TW06 sphere-derived cells were grown in agar and various doses of EGCG were added for 2 weeks. Colonies were counted at the end of the incubation period, and we found that EGCG inhibited the growth of colonies in a dose-dependent manner (Figure 5). The results suggest that EGCG can inhibit the self-renewal and tumorigenicity capacity of NPC CSC.

**Figure 6:**
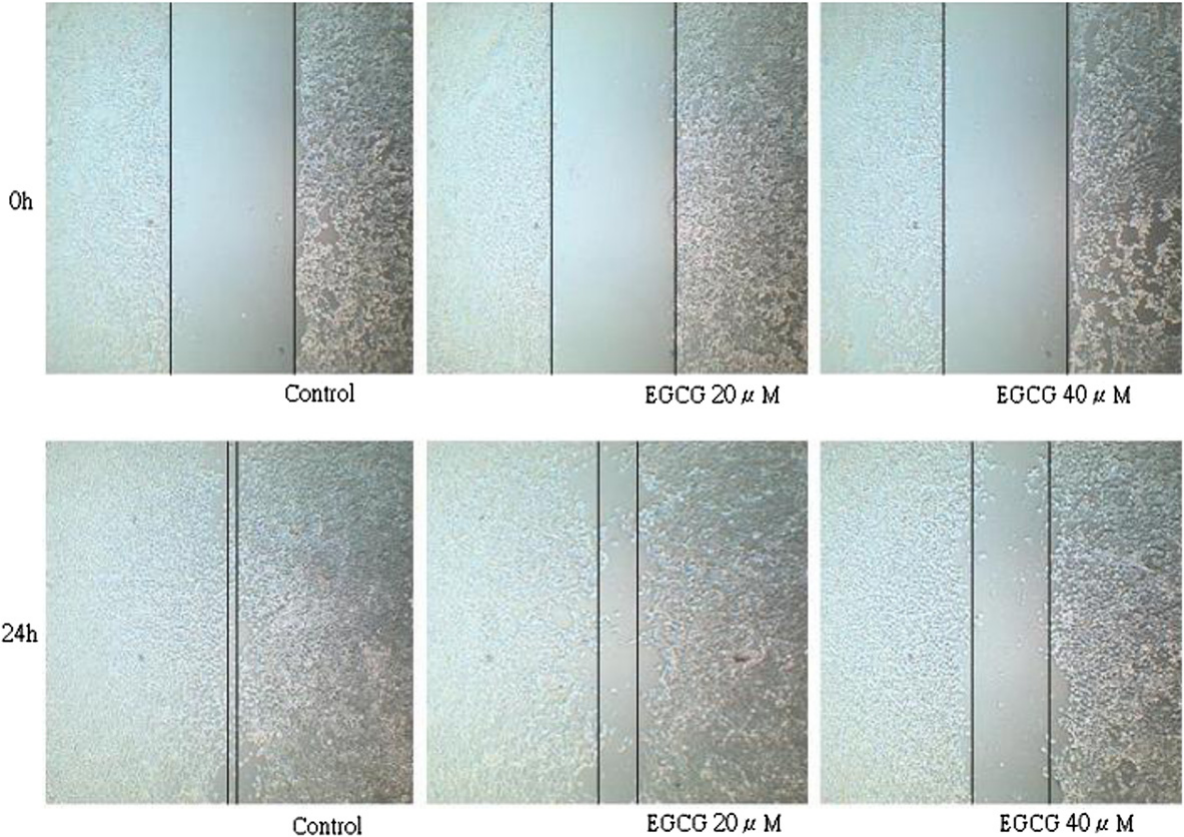
**EGCG inhibited cell migration shown by wound healing assay.** TW01 sphere-derived cells produced marked cell migration in the wound area 24 h after wounding, but wounds treated with EGCG showed significant delays under the same conditions.

**Figure 7:**
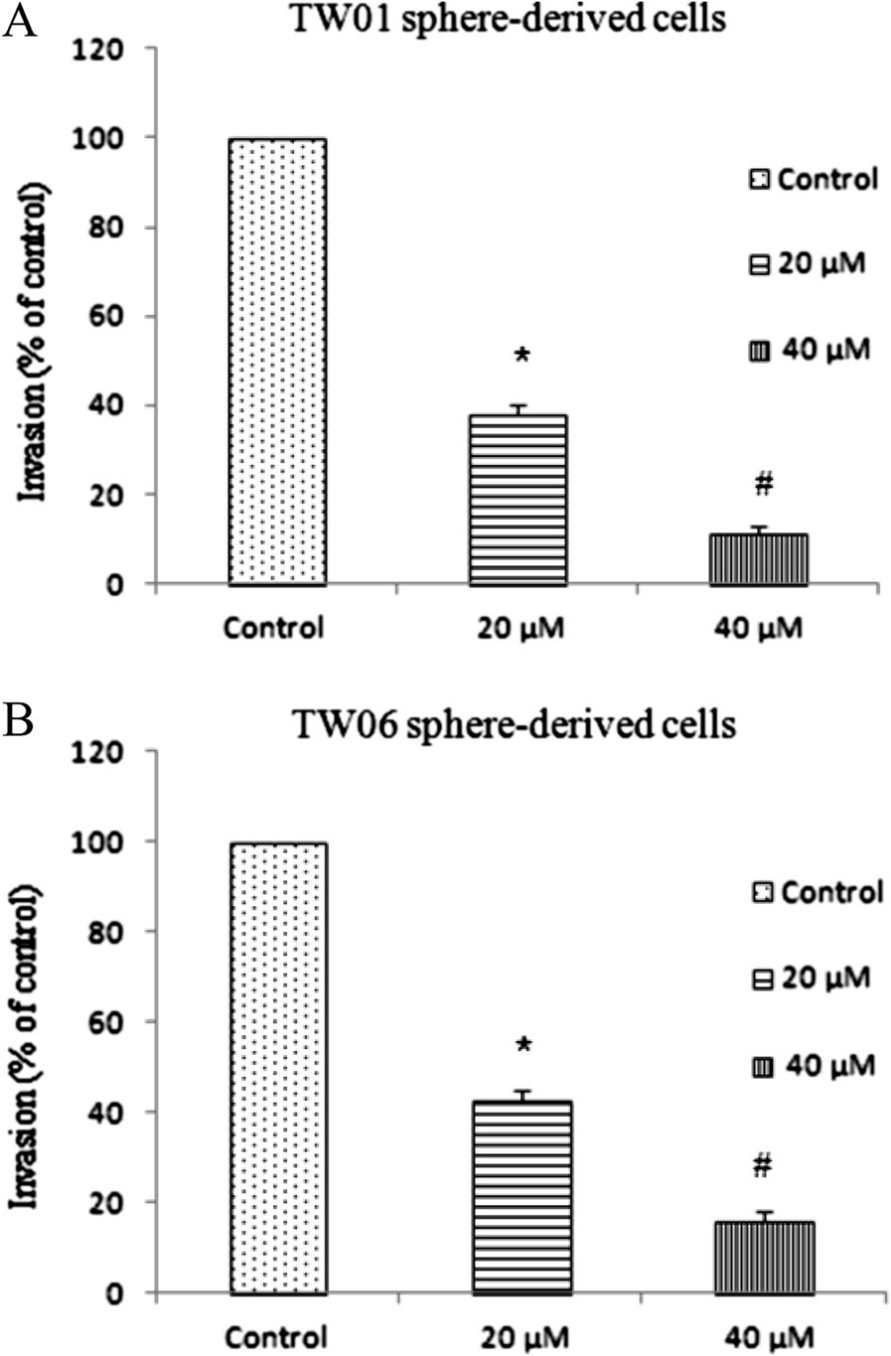
**Inhibition of cell invasion of EGCG in a dose-dependent manner.** EGCG inhibits invasion abilities of NPC sphere-derived cells. (**A**) TW01 sphere-derived cells, (**B**) TW06 sphere-derived cells. Data represent mean ± SD. * or # were significantly different from respective controls, *p* < 0.05.

Wound-healing assay was performed to assess whether EGCG affected TW01 sphere-derived cell migration. The control group without EGCG treatment produced marked cell migration in the wound area 24 hr after wounding, but wounds treated with EGCG showed significant delays under the same conditions (Figure 6). By the cell invasion assay, the invasive ability was significantly inhibited in sphere-derived cells treated with EGCG in a dose-dependent manner (Figure 7).

### EGCG regulated the expression of stem cell genes in sphere-derived cells

**Figure 8:**
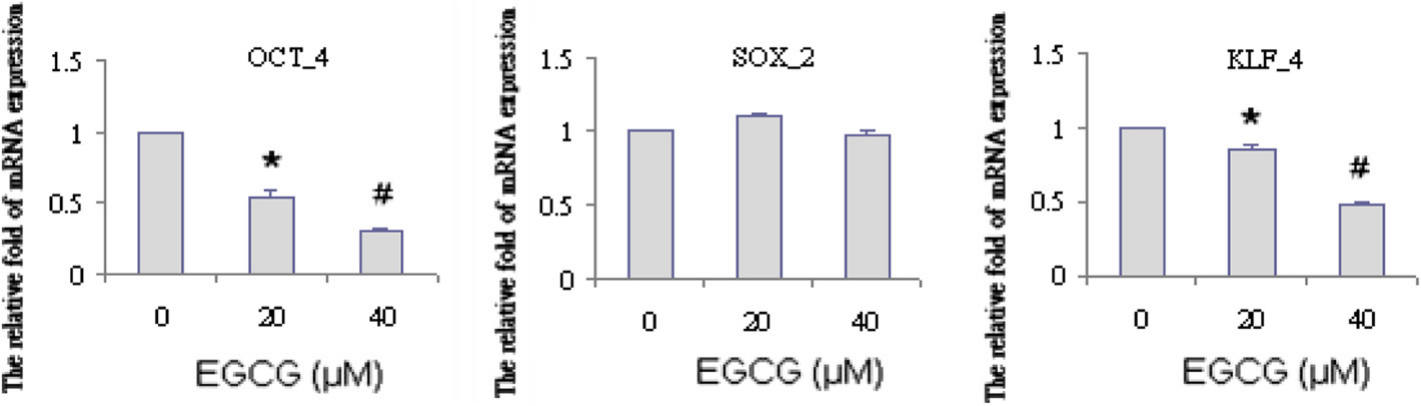
**Expression of stemness genes was blocked by EGCG.** The expression of stem cell genes (Oct4, Sox2, Klf4) of TW01 sphere-derived cells treated with EGCG was measured by the q-RT-PCR. Decreasing expression of OCT4 and Klf4 was accompanied with EGCG dosage. Data is represented mean ± SD. * or # means significantly different from respective controls, *p* < 0.05.

Increased expression of stemness genes--Sox-2, Klf-4, and Oct-4 was detected in sphere-derived cells compared to parental cells. We next examined the effects of EGCG on the mRNA expression levels of these factors. TW01 sphere-derived cells were treated with or without EGCG. After a 36 h incubation period, cells were harvested and the expression of Sox-2, Klf-4, and Oct-4 was measured by the real-time RT-PCR (Figure 8). The results showed EGCG inhibited the expression of Klf-4 and Oct-4 in sphere-derived cells. However, the expression of Sox-2 was not affected by EGCG.

### EGCG regulated the expression of EMT-related protein

**Figure 9:**
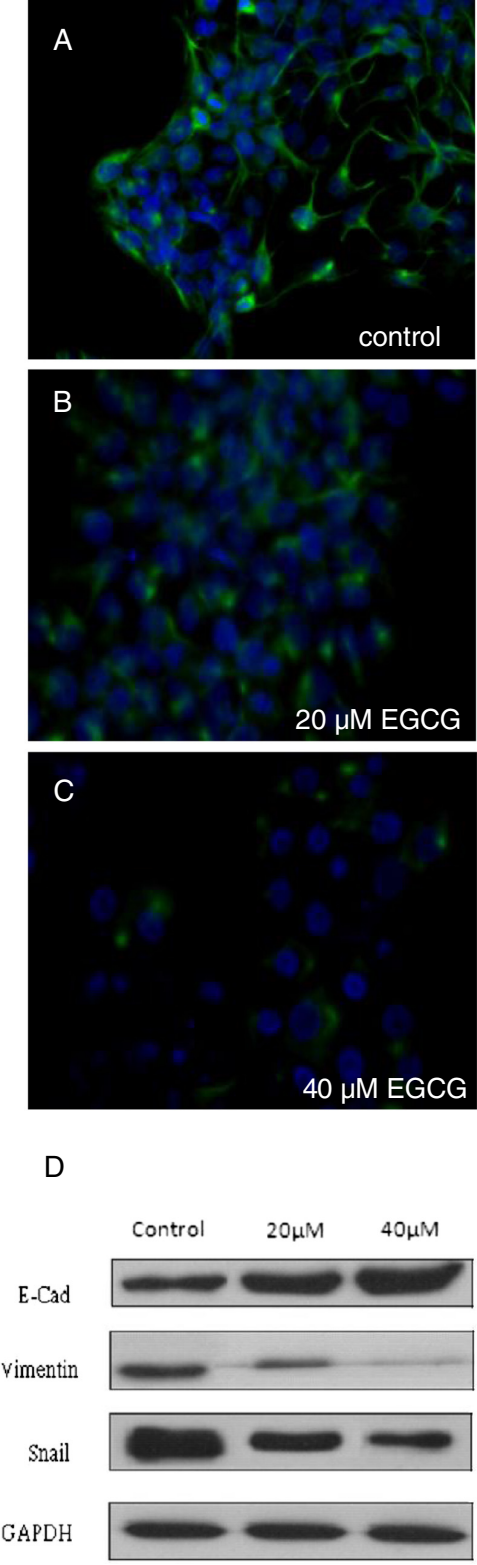
**Effect of EGCG on EMT phenotype.** Mesenchymal cell marker, vimentin expression was assayed by immunofluorescence staining in TW01 sphere-derived cells (**A**~**C**). (**A**) control group (**B**) treated with 20 μM EGCG (**C**) treated with 40 μM EGCG. EGCG could decrease the expression of vimentin. Green fluorescent signal represents vimentin expression. Blue color is DAPI stain for nucleus. (**D**) Western blot signals in TW01 sphere-derived cells show decreased expression of vimentin and snail and increased expression of E-cadh (e-cadherin) after EGCG treatment.

Due to change of cell shape and EMT marker expression, NPC sphered cells might have undergone EMT change. We then studied whether the expression of EMT markers regulated by EGCG in TW01 sphere-derived cells correlated with migration and invasive property changes. Immunofluorescence staining in sphere-derived cells treated with or without EGCG was performed and showed that vimentin expression was decreased in EGCG-treated cells (Figure 9A~C). Moreover, in western blotting, EGCG significantly inhibited the levels of Snail, Vimentin and increased E-Cadherin expression in a dose-dependent manner (Figure 9D). Our results showed that the invasion and migration ability of TW01 sphere-derived cells was inhibited by EGCG in relation to regulating EMT-related protein expression.

## Discussion

Unlimited growth, recurrence, and metastasis of cancer have been related to the behavior of CSC that can be identified by *in vitro* assay. Cancer stem cells undergoing metastasis that have been demonstrated to have EMT marker expressions associated with invasive and migratory properties easily invade the surrounding tissues. This invasive type of cancer cell acquired mesenchymal, fibroblast-like morphology and shows reduced intercellular adhesion and increased motility [12]. Tumor progression is frequently associated with the down-regulation of E-cadherin, and up-regulation of vimentin, and several transcription factors including Snail, Twist and Slug [12,25]. A major clinical feature of NPC is frequent involvement of regional lymph nodes and distant organ metastasis. Even after treatment, increased invasiveness and migration of NPC during metastasis leads to poor outcomes and increased patient mortality [26]. Previous studies showed EMT molecular events associated with NPC metastasis were obvious in the absence of the Epstein-Barr virus genome [27].

The isolation of CSC from cancer cells was achieved successfully using several techniques. CSC can be enriched in spheres cultured in serum-free mediums supplemented with mitogens, such as the basic fibroblast growth factor (bFGF) and epidermal growth factor (EGF) [13,28,29]. Such cancer cells grown in a suspended condition exhibit resistance to anoikis, resulting in acquisition of ability to survive and proliferate [30].

We quantified and enriched CSC within NPC cell lines and subsequently characterized their phenotypical and functional properties, such as invasion capacity and epithelial-mesenchymal transition (EMT). These spheres contain putative CSC and can provide niches for the maintenance and growth of cancer cells. The EMT-type cells share many biological characteristics of CSC, which are linked closely with tumor recurrence and metastasis.

Previous studies exploited EGCG as a potential agent for prevention and treatment of cancers. EGCG suppresses the expression of HSP70 and HSP90, and exhibits anti-tumor activity in vitro and in vivo [31]. Moreover, in addition to EGCG-induced apoptosis effects, previous researches have recognized that EGCG-induced p21, p53, p16, and p27 expression, which is associated with negative regulations of cell cycle progression [32-34]. However, there were limited reports about EGCG regulation effects on CSC. Our study proposes phenotype changes and functional consequences, by which the inhibitory effects of EGCG on CSC properties of NPC are seen.

Our designed experiments recognized the effects of EGCG on isolated sphere cells with anchorage-independent growth, and obtained novel findings. We discovered EGCG inhibits growth and induces apoptosis in parental NPC cells, but is inefficient in sphere-derived cells. Relative chemo-resistant NPC cells in sphere-derived cells, in accordance to Chen’s study on non-adhesive culture systems exhibiting CSC characteristics, can be used in cancer research [35].

On the other hand, EGCG can inhibit sphere formation and regulate invasive-like phenotypes characterized by EMT of cells grown in serum free non-adherent cultures. In addition, it inhibited colony formation of sphere-derived cells grown in agar in a dose-dependent manner. This implicated that EGCG can be effective in suppressing the self-renewal capacity of NPC CSC. Furthermore, at the mRNA level, the expression of stemness maintaining transcription factors Klf-4 and Oct-4 were inhibited by EGCG. Several studies suggested Klf-4 and Oct-4 play important roles in maintaining stem cell identity, and are responsible for increased cell migration and invasion [36,37]. Our study further showed that EGCG inhibits the factors required for maintaining the self-renewal capacity in NPC CSC.

Because CSCs appear to have a significant role in invasion and metastasis, we measured the effects of EGCG on invasion and migration of NPC sphere-derived cells. The results showed that invasive and migratory behaviors were significantly reduced in the EGCG-treated group in a dose-dependent manner. This implied EGCG efficiently suppressed the metastasis ability in NPC CSC. Recent studies have also described the inhibitory activities of EGCG targeting cancer stem cells, which can suppress CSC self-renewal properties and block their migration and invasion [38,39].

Enhanced EMT characteristics are associated with poor outcomes and declined survival in patients with NPC [40]. We examined EGCG regulating translational levels of EMT expression by TW01 sphere-derived cells. The western blot result showed EGCG inhibits the expression of vimentin, Snail, and is accompanied by the upregulation of E-cadherin. Immunofluorescence staining showed that vimentin expression in sphere-derived cells was decreased in the EGCG group compared to the control group. These results showed that EGCG may block NPC early metastasis signaling. Dysregulation of E-cadherin and β-catenin functions in cell-cell adhesion is common in NPC, and correlates with advanced stage disease and lymph node metastasis [41]. Our results further showed that EGCG inhibited NPC invasion and migration, correlating with the elevation of E-cadherin levels in NPC sphere-derived cells. E-cadherin is an important cell adhesion molecule, and has a key role in the early stage of tumor metastasis. Further investigation is required to study how EGCG modulates the expression of E-cadherin proteins.

Research regarding the use of EGCG against cancer has been proposed, however, most concentrations of EGCG used in cell lines are higher than the plasma concentrations observed in people after consuming tea because of its limited bioavailability *in vivo*[42]. To increase bioavailability and stability of EGCG, particular studies have elucidated structure-modified compounds with higher efficacy, which also possess anticancer activities [39,43]. Previous research has shown evidence of EGCG-induced apoptosis and cytotoxicity specifically on cancer cells, but not on normal cells under the same EGCG treatment [44,45]. This suggests that EGCG serves as an acceptable safety agent.

Overall, our findings further clarify the anti-cancer effects of EGCG, which can eliminate the stem-cell characteristics of NPC, and inhibit epithelial-mesenchymal transition (EMT), partially because of regulated EMT-related proteins and lessen the migratory and invasive abilities of NPC. EGCG may be potentially effective in preventing the recurrence and metastasis of NPC in combination with standard treatment.

## Conclusions

Our data shows that NPC cells with sphere formations possess the properties of CSC including EMT signatures, and their self-renewal capacities and invasive characteristics are inhibited by EGCG. This data supports the pivotal role of EGCG as a dietary compound targeting NPC, and may decrease recurrence and metastasis in nasopharyngeal carcinoma cells.

## Abbreviations

EGCG: Epigallocathechin gallate; CSC: Cancer stem-like cell; TIC: Tumor-initiating cell; NPC: Nasopharyngeal carcinoma; EMT: Epithelial-mesenchymal transition; MTT: 3-(4,5-dimethylthiazol-2-yl)-2,5-diphenyltetrazolium bromide; MMP: Matrix metalloproteinases; BME: Basement membrane extract.

## Competing interests

The authors have declared no conflict and financial competing of interests.

## Authors’ contributions

CHL: designed the study, performed experimental works, analyzed data, and generated the figures and manuscript. YAS: designed the protocol for NPC CSC selection and sphere formation. PHH: data analysis and performed the statistical analysis. YBY: intellectual support and coordination of the study. YJC: Project design, project coordination and manuscript preparation. All authors have read and approved of the final version of the manuscript.

## Pre-publication history

The pre-publication history for this paper can be accessed here:

http://www.biomedcentral.com/1472-6882/12/201/prepub
